# Emerging roles of protein kinase CK2 in abscisic acid signaling

**DOI:** 10.3389/fpls.2015.00966

**Published:** 2015-11-03

**Authors:** Belmiro Vilela, Montserrat Pagès, Marta Riera

**Affiliations:** Centre for Research in Agricultural Genomics (CRAG), CSIC-IRTA-UAB-UB Consortium, Campus UAB, Barcelona, Spain

**Keywords:** protein kinase CK2, ABA signaling, proteasome degradation, circadian clock, post-translational modifications

## Abstract

The phytohormone abscisic acid (ABA) regulates many aspects of plant growth and development as well as responses to multiple stresses. Post-translational modifications such as phosphorylation or ubiquitination have pivotal roles in the regulation of ABA signaling. In addition to the positive regulator sucrose non-fermenting-1 related protein kinase 2 (SnRK2), the relevance of the role of other protein kinases, such as CK2, has been recently highlighted. We have recently established that CK2 phosphorylates the maize ortholog of open stomata 1 OST1, ZmOST1, suggesting a role of CK2 phosphorylation in the control of ZmOST1 protein degradation (Vilela et al., 2015). CK2 is a pleiotropic enzyme involved in multiple developmental and stress-responsive pathways. This review summarizes recent advances that taken together suggest a prominent role of protein kinase CK2 in ABA signaling and related processes.

## Introduction

The phytohormone abscisic acid (ABA) plays a central role in plant development and responses to abiotic stress (Leung and Giraudat, 1998; Finkelstein et al., 2002). Water stress conditions induce the accumulation of ABA levels in guard cells and this increase promotes the closing of stomata in order to reduce transpiration and water loss (Schroeder et al., 2001). The molecular mechanism of ABA action is now well-established in *Arabidopsis* (Cutler et al., 2010; Kim et al., 2010; Klingler et al., 2010; Raghavendra et al., 2010; Umezawa et al., 2010; Zhang et al., 2015). ABA triggers downstream responses by binding to the cytosolic receptors pyrabactin resistance/pyrabactin-like/regulatory component of ABA receptor (PYR/PYL/RCAR), which then sequester the negative regulators clade A type 2C protein phosphatases (PP2C), allowing the activation of Group III Sucrose non-fermenting-1 related protein kinases 2 (SnRK2; Ma et al., 2009; Park et al., 2009). These three protein types are necessary and sufficient to mediate an ABA triggered model signaling cascade *in vitro* (Fujii et al., 2009). Recent advances engineering ABA receptors using agrochemicals open new possibilities for crop improvement (Park et al., 2015).

Reversible protein phosphorylation is therefore a key protein modification involved in ABA signaling and it allows for the rapid regulation of protein function. In addition to the central role of Group III SnRK2s, multiple kinases have been implicated in ABA signaling. Calcium-dependent protein kinases (CDPKs) function as calcium sensors and are hub regulators of Ca^2+^-mediated immune and stress responses (Mori et al., 2006; Boudsocq and Sheen, 2013). CBL-interacting protein kinases (CIPKs), another family of kinases involved in calcium signaling, regulate potassium transport processes in roots and in stomatal guard cells (Cheong et al., 2007). Moreover, mitogen activated protein kinases (MAPKs) are induced by ABA to elicit a stress response (Danquah et al., 2015).

There is growing amount of data linking protein kinase CK2 to ABA signaling and abiotic stress responses, as shown in this review. CK2 is an evolutionary conserved Ser/Thr kinase found in all eukaryotes. The CK2 holoenzyme is a heterotetramer composed by two types of subunits, two catalytic (CK2α) and two regulatory (CK2β; Litchfield, 2003). Unlike animals, in plants both kinds of subunits are encoded by multigenic families (Velez-Bermudez et al., 2011). Plant CK2 is a pleiotropic enzyme involved in relevant processes such as plant growth and development, light-regulated gene expression, circadian rhythm, hormone responses, cell-cycle regulation, flowering time, DNA repair or responses to biotic and abiotic stress, among others (Riera et al., 2013; Mulekar and Huq, 2014).

## Role of Protein Kinase CK2 in ABA Signaling

Since CK2 is essential for plant viability and the depletion of CK2α is lethal, as previously demonstrated in yeast (Padmanabha et al., 1990), plant genetic approaches involving CK2 have been difficult. The first *Arabidopsis* CK2α antisense plants produced confirmed the role of CK2 in light-regulated gene expression and plant growth (Lee et al., 1999). In recent years, several transgenic lines for CK2α have been generated. An inducible dominant-negative for CK2α plants evidenced that CK2 control chloroplast development, cotyledon expansion, root and shoot growth, as well as altered cell division, cell expansion and auxin transport (Moreno-Romero et al., 2008; Marquès-Bueno et al., 2011). *Arabidopsis* mutated for all three nuclear CK2α subunits (α1α2α3) or doubly mutated in all possible combinations, show a significant decrease of CK2 activity, and a clear phenotype of late flowering. This indicates that that CK2α subunits influence the circadian clock period of oscillation (Lu et al., 2011). Moreover, CK2α *knockout* lines display altered developmental and stress responsive pathways with a marked hyposensitivity to ABA and high salt when tested by the criteria of seed germination and cotyledon greening (Mulekar et al., 2012).

Chloroplastic isoforms of CK2α (cpCK2α) have been identified in most higher plants (Turkeri et al., 2012; Vélez-Bermúdez et al., 2015). Different phosphoproteomic approaches in *Arabidopsis* demonstrate the prominent role of cpCK2 for phosphorylation in these organelles (Reiland et al., 2009; Schonberg et al., 2014). ABA affects the transcription of most chloroplastic genes (Yamburenko et al., 2013, 2015). Mutation of chloroplastic isoform CKA4 in *Arabidopsis* gives a phenotype of reduced sensitivity to ABA during seed germination and seedling growth, and increased stomatal aperture and leaf water loss (Wang et al., 2014). These effects were attributed to the downregulation of ABA-responsive genes, including OST1, a representative SnRK2 kinase central to ABA signaling. The same work suggests that CK2 is involved in retrograde signaling from chloroplast to nucleus, since the expression levels of the transcription factor *ABI4*, directly involved in retrograde and ABA signaling, were reduced in the *cka4* mutant under ABA treatment (León et al., 2012). Recent work analyzing CK2A4 RNAi lines in the CK2α triple mutant background confirmed the importance of this gene in the regulation of ABA response, lateral root formation and flowering time, in a process that could be regulated by retrograde signaling (Mulekar and Huq, 2015).

Even though more that 300 substrates have been described for mammalian CK2 (Meggio and Pinna, 2003; Bian et al., 2013), the confirmed number of CK2 plant substrates is lower, around 50, as shown in Table 1. Among these substrates, CK2 phosphorylation of maize LEA protein RAB17 has been one of more extensively characterized examples (Plana et al., 1991). LEA proteins/RAB/dehydrins accumulate during embryogenesis and their protein level correlates with increased levels of ABA and acquisition of desiccation tolerance (Galau et al., 1986; Ingram and Bartels, 1996). Previous work performed in our group established that CK2 phosphorylation regulates the intracellular dynamics and subcellular localization of maize RAB17. The phosphodeficient mutant form of RAB17, when overexpressed in transgenic *Arabidopsis*, leads to a failure of seed germination arrest in osmotic stress conditions (Plana et al., 1991; Riera et al., 2004). The homologs of Rab17 in tomato (TAS14) and in *Arabidopsis* (ERD14) are also phosphorylated by CK2 (Godoy et al., 1994; Alsheikh et al., 2003). Other dehydrins as TsDHN1, 2 from *Thellungiella salsuginea* can stabilize the cytoskeleton under stress conditions, in a process that may involve CK2 phosphorylation (Rahman et al., 2011). Recently, ZmLEA5C that enhances tolerance to osmotic and low temperature stresses in transgenic tobacco and yeast has been also described as a CK2 substrate (Liu et al., 2014). Different types of transcription factors are also CK2 substrates, some of them involved in ABA response, as EmBP-2 and ZmBZ-1. These two b-ZIP transcription factors are phosphorylated by CK2 and this modification alters their DNA binding capacity (Nieva et al., 2005). Also OREB1, a rice ABRE binding factor is phosphorylated by multiple kinases such as SnRK2 and CK2 (Hong et al., 2011). These factors bind to ABRE (ABA Responsive Elements) in the nucleus and activate the transcription of ABA-inducible genes, suggesting that CK2 regulation of RAB proteins could involve not only direct phosphorylation but also altered gene expression.

**TABLE 1:** **List of plant CK2 substrates**.

**Name**	**Type**	**Species**	**Role**	**References**
**Light-signal transduction pathway and circadian clock**				
AT-1	DNA binding factor	Pea	Binds to ATI-box elements in light regulated promoters	Datta and Cashmore (1989)
ATBP-1	DNA binding factor	Pea	Binds to ATI-box elements in light regulated promoters	Tjaden and Coruzzi (1994)
GBF1	bZIP TF	*Arabidopsis*	Binds to G-box elements in light regulated promoters	Klimczak et al. (1995)
Opaque2	bZIP TF	Maize	Circadian clock regulated	Ciceri et al. (1997)
CCA1	Myb-related TF	*Arabidopsis*	Circadian clock regulator	Sugano et al. (1998)
LHY, OsLHY	Myb-related TF	*Arabidopsis*, Rice	Circadian clock regulator	Sugano et al. (1998); Ogiso et al. (2010)
HY5	bZIP TF	*Arabidopsis*	Promotes photomorphogenesis	Hardtke et al. (2000)
HFR1	bHLH TF	*Arabidopsis*	Promotes photomorphogenesis	Park et al. (2008)
PIF1	Phytochrome interacting factor	*Arabidopsis*	Represses photomorphogenesis	Bu et al. (2011)
**Abiotic and biotic stress**				
ZmSnRK2/ZmOSTl	Protein kinase	Maize	ABA signaling	Vilela et al. (2015)
Rabl7,ZmLEA5cERD14, TAS-14	LEA proteins	Maize, *Arabidopsis*, tomato, wheat	Stress responsive proteins	Plana et al. (1991); Liu et al. (2014);Alsheikh et al. (2003); Godoy et al. (1994)
TsDHNl,2	Dehydrins	*Thellungiella salsuginea*	Stress responsive proteins	Rahman et al. (2011)
EmBP-2/ZmBZ-l	bZIP TF	Maize	Activates transcription of the abscisic acid-inducible gene rab28	Nieva et al. (2005)
TGA2	bZIP TF	*Arabidopsis*	Binds to promoter of salicilic-induced genes	Kang and Klessig (2005)
OREB1	ABRE binding factor	Rice	Binds to ABRE (ABA responsive Elements)	Hong et al. (2011)
p23	co-chaperone protein	*Arabidopsis*	Plant response to Salicihc acid	Tosoni et al. (2011)
PCS	phytochelatin synthase	*Arabidopsis*	Synthesis of heavy metal-binding peptides	Wang et al., 2009
**Chromatin associated and nuclear proteins**				
lamin-like protein	lamina matrix protein	Pea	Nuclear stability, chromatin organization	Li and Roux (1992)
MFP1	coil-coil protein	Tomato *Allium cepa*	Structural roles in nuclear matrix and chloroplast	Meier et al. (1996); Samaniego et al. (2006)
NopA64/nopA61	nucleolin-like phosphoproteins	*Allium cepa*	Located in nucleolus	de Cárcer et al. (1997)
P-proteins	Ribosomal proteins	Maize	Complex with 60S ribosomal subunits	Bailey-Serres et al. (1997)
DNA helicase I	DNA helicase I	Pea	DNA transcription	Tuteja et al. (2001)
DNA topoisomerase I	DNA topoisomerase I	Pea	DNA transcription	Tuteja et al. (2003)
HMGB proteins	High mobility group B proteins	Maize, *Arabidopsis*	Chromatin associated proteins	Stemmer et al. (2002)
SSRP1	structure-specific recognition protein	Maize	Chromatin associated proteins	Krohn et al. (2003)
eIF2ab/3c/4b/5	elongation initiation factors	*Arabidopsis*, maize, wheat	Translation initiation	Dennis and Browning (2009)
Histone deacetilase 2B	Histone deacetilase	*Arabidopsis*	Chromatin remodeling enzyme	Dennis and Browning (2009)
**Chloroplast machinery**				
Chloroplast RNPs/28RNP/p34/RNP29,33	Ribonucleoproteins	Spinach, *Arabidopsis*	RNA binding proteins involved in chloroplast RNA processing and stabilization	Kanekatsu et al. (1993, 1995); Lisitsky and Schuster (1995); Reiland et al. (2009)
CP29	photosystem II subunit	Maize	Light harvesting complex import	Testi et al. (1996)
TOC159	preprotein receptor	*Arabidopsis*	Nuclear-encoded chloroplast preproteins from the cytosol	Agne et al. (2010)
SIG1/SIG6	plastid sigma factors	*Arabidopsis*	Gene-regulatory proteins for promoter binding and transcription initiation	Schweer et al. (2010)
Alb3	Thylakoid membrane protein	*Arabidopsis*	Thylakoid biogenesis	Schonberg et al. (2014)
**Other**				
CFOCFl-ATPase	Chloroplast ATP synthase (b subunit)	Spinach	ATP synthesis	Kanekatsu et al. (1998)
C2	subunit of the 20S proteasome	Rice	Protein degradation of ubiquitinated proteins	Umeda et al. (1997)
gpl00/gp96	Glycyrrhizin (GL)-Binding Protein (gp100)	Soybean	Lipoxygenase that catalyzes the oxygenation of unsaturated fatty acids	Ohtsuki et al. (1994, 1995)
β-Conglycinin α Subunit	β-Conglycinin α Subunit	Soybean	storage protein	Ralet et al. (1999)
calreticulin	Calreticulin	Spinach	Ca^2+^ binding protein	Baldan et al. (1996)
apyrase	apyrase	Pea	ATP hydrolysis	Hsieh et al. (2000)

We have recently established the maize ortholog of open stomata 1 OST1 (also known as SnRK2.6 or SnRK2E) as a phosphorylation target of CK2 (Vilela et al., 2015). CK2 phosphorylates ZmOST1 at a cluster of serines in the ABA box with implications on protein levels, kinase activity, and response to abiotic stimuli. Transgenic *Arabidopsis* plants overexpressing ZmOST1 mutagenized at CK2 phosphorylation sites are more resistant to drought and are hypersensitive to ABA at the level of stomata.

## ABA Signaling and Proteasome Degradation

In addition to phosphorylation, other post-translational modifications such as ubiquitination, and sumoylation play significant roles in regulating ABA signaling (Lyzenga and Stone, 2012). Ubiquitination of the PYR/PYL/RCAR ABA receptors causes their degradation in the absence of ABA (Irigoyen et al., 2014). DDB1-ASSOCIATED1 (DDA1), a protein part of the CULLIN4-RING E3 ubiquitin ligase, binds to PYR8, PYL4 and PYL9 and facilitates their proteasomal degradation, negatively regulating ABA responses. Conversely, ABA protects PYL8 from destabilization by limiting its polyubiquitination by a process that is still unknown. ABA also reduces PYL8 expression after 3h of treatment in a process that would facilitate a faster receptor turnover, after the signal is attenuated (Irigoyen et al., 2014). In addition, the turnover of PYL4 and PYR1 in the proximity of the plasma membrane is regulated by the interaction with a single subunit RING-type E3 ubiquitin ligase, RSL1 (Bueso et al., 2014).

Several transcription factors involved in ABA signaling as ABI3, ABI5, ABFs ABI4, and ATHB6 can also be regulated by proteasome degradation. The B3-domain transcription factor ABSCISIC ACID-INSENSITIVE 3 (ABI3), a central regulator in ABA signaling, is an unstable protein that is polyubiquitinated by an ABI3-interacting protein (AIP2), which contains a RING motif. AIP2 negatively regulates ABA signaling by targeting ABI3 for post-translational destruction (Zhang et al., 2005). During vegetative growth, ABA induces AIP2 expression, tightly regulating ABI3 turnover while promoting its accumulation during seed maturation. Another example is ABSCISIC ACID INSENSITIVE 5 (ABI5), a member of the basic leucine zipper (bZIP) transcription factor, that plays an important role in controlling ABA dependent postgerminative growth arrest as well as late phases of seed maturation (Finkelstein and Lynch, 2000; Lopez-Molina et al., 2001). The abundance of ABI5 is tightly controlled by the ubiquitin-*26S* proteasome system. KEEPONGOING (KEG), a RING3-type E3 ubiquitin ligase, negatively regulates ABA signaling by promoting ABI5 ubiquitination and subsequent degradation by the *26S* proteasome (Liu and Stone, 2010). This process occurs in the cytosol when ABA is absent (Liu and Stone, 2013). In the nucleus, ABI5 stability is regulated by another negative regulator of ABA, a E3 ubiquitin ligase assembled with ABA-hypersensitive DCAF1 (ABD1; Seo et al., 2014). In addition to ubiquitination, sumoylation of ABI5 is thought to maintain a degradation-resistant inactive pool of ABI5 in the absence of ABA (Miura et al., 2009). An additional class of ABI5-interacting proteins, the AFPs, has also been reported to alter ABI5 stability (Lopez-Molina et al., 2003). Another group of positive effectors in ABA responses regulated by proteasome degradation is the ABA Binding Factor/ABA-Responsive Element Binding Proteins (ABF/AREB) subfamily of bZIP-type transcription factors. ABF1 and ABF3 have similar functions to ABI5 in regulating seed germination and post-germinative growth (Finkelstein et al., 2005). ABF1 and ABF3 are ubiquitylation substrates of KEG and the abundance of both proteins is affected by ABA and the ubiquitin pathway (Chen et al., 2013). The stabilization of ABF1 and ABF3 by ABA is thought to be achieved by phosphorylation by SnRK2 kinases, which in turn promotes the binding of 14–3–3 proteins (Sirichandra et al., 2010). ABSCISIC ACID INSENSITIVE 4 (ABI4), a member of the DREB subfamily A-3 of ERF/AP2 transcription factors, is required for proper ABA signaling during seed development and germination (Gregorio et al., 2014). Like ABI3 and ABI5, ABI4 is subject to a stringent post-transcriptional regulation that targets the protein to degradation and prevents it from accumulating to high levels. However, unlike ABI3 and ABI5, ABI4 is not stabilized in the presence of ABA (Finkelstein et al., 2011). Finally, the HD-Zip transcription factor ATHB6 physically interacts with the PP2C phosphatase ABI1 and it has been described as a negative regulator of the ABA signal pathway (Himmelbach et al., 2002). Moreover, ABA negatively regulates ATHB6 protein turnover (Lechner et al., 2011).

**FIGURE 1:**
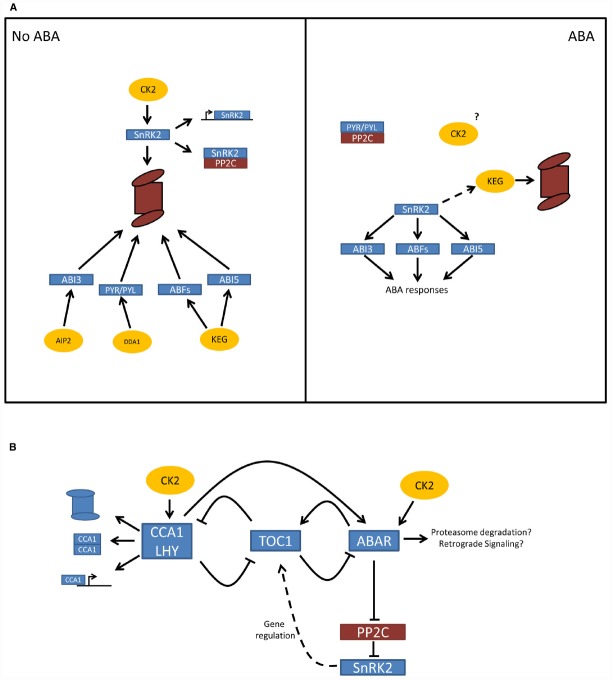
**(A)** Regulation of ABA signaling by the control of protein turnover. In the absence of ABA, major regulators of the hormone such as the PYR/PYL/RCAR receptors, the SnRK2 kinases, and several transcription factors (ABI3, ABI5, ABF1, ABF3) are degraded by the proteasome, and/or inactivated. In this way the output of the ABA signal is thoroughly dampened in the absence of the hormone. When ABA levels rise, these major regulators are protected from degradation through the inactivation or degradation of the negative regulators such as KEG. CK2 is known to mediate the stabilization and destabilization of proteins in other systems and is a likely candidate to also have a role as a housekeeping kinase controlling protein turnover in ABA signaling. **(B)** Integration of the plant circadian clock on ABA signaling. The core circadian clock consists of a negative feedback loop between the CCA1 and LHY on one hand and TOC1 on the other. ABA treatment induces TOC1 expression and, in another feedback loop, TOC1 attenuates ABA signaling and negatively regulates the expression of ABA signaling genes like ABAR, known to interact with the ABA central signaling complex. CCA1 an LHY act synergistic to ABA and antagonistic to TOC1 expression. CCA1 and LHY are phosphorylated by CK2 targeting them for degradation, promoting CCA1 dimerization, CCA1-DNA complex formation, and interaction with the promoters of downstream genes. ABAR is also a substrate of CK2 and, even though the effects of this activity are still unknown, they could include protein turnover and altered gene expression by retrograde signaling from the chloroplast.

Proteosomal degradation in response to ABA is regulated by phosphorylation/dephosphorylation mechanisms (Figure 1A). For instance, ABA promotes the self ubiquitination and degradation of KEG after phosphorylation, a process that could be regulated by the SnRK2 kinases belonging to the core ABA signaling complex (Antoni et al., 2011). Another kinase, Calcineurin B-like Interacting Protein Kinase 26 (CIPK26) interacts with the ABA signaling components ABI1, ABI2, and ABI5. CIPK26 influences the sensitivity of germinating seeds to the inhibitory effects of ABA and is also targeted by KEG for proteasomal degradation (Lyzenga et al., 2013).

Our recent work points toward a role of protein kinase CK2 in control of ZmOST1 protein degradation (Vilela et al., 2015). CK2 phosphorylation enhances ZmOST1 interaction with PP2C phosphatases, probably causing a sustained “off” state of kinase activity, and also primes SnRK2 for protein degradation through the *26S* proteasome pathway. Thus, CK2 seems to act in dampening the ABA signal output through its action on ZmOST1 while at the same time inducing ZmOST1 transcription (Figure 1A). This type of regulation would be particularly effective in the absence of ABA, with the silencing of SnRK2 output and the preparation of the new state of ABA response.

## Other Implications of CK2 Action in ABA Signaling

One particularly important process in the regulation of plant-water relationship is the incorporation of circadian responses in the output of the ABA signal. In fact, the regulation of circadian rhythms to anticipate daily and seasonal environmental cycles allows the plant to optimally incorporate external conditions into internal processes. Stomata, for instance, are able to anticipate the dawn and dusk signals, and are more responsive to ABA in the afternoon, coinciding with the timing of (Ca^2+^) peak oscillations (Seo and Mas, 2015).

Circadian rhythms are autoregulatory, endogenous rhythms with a period of approximately 24 h. In *Arabidopsis*, the core circadian clock is made up of genes that interact through a series of transcriptional and post-transcriptional feedback loops to create rhythmic gene expression (Seo et al., 2012; Bendix et al., 2015). Briefly, the core circadian clock consists of a negative feedback loop between the two homologous MYB-like transcription factors CIRCADIAN CLOCK ASSOCIATED 1 (CCA1) and LATE ELONGATED HYPOCOTYL (LHY) on one hand and TIMING OF CAB EXPRESSION1 (TOC1) on the other (Fogelmark and Troein, 2014).

TOC1 and ABA work antagonistically to achieve the optimal response to water status. ABA treatment induces TOC1 expression and, in a feedback loop, TOC1 attenuates ABA signaling and negatively regulates the expression of ABA signaling genes. TOC1 mis-expressing plants have defects in ABA-dependent stomata closure and altered tolerance to drought stress (Legnaioli et al., 2009). Consequently, CCA1 an LHY should be synergistic to ABA and antagonistic to TOC1 expression (Pokhilko et al., 2013). Interestingly, one of the ABA genes negatively regulated by TOC1 is the magnesium quelatase subunit H (ABAR/CHLH/GUN5). ABAR is involved in retrograde signaling and positively regulates guard cell signaling in response to ABA. It has been recently demonstrated that ABAR and OST1 can interact *in vitro*, but that ABAR phosphorylation is independent of OST1 since it apparently acts upstream of the PP2C-SnRK2 complex (Liang et al., 2015). It should be noted that ABAR has been suggested as a potential substrate of cpCK2 (Reiland et al., 2009; Schonberg et al., 2014) but additional experiments are required to elucidate the effect of CK2 activity on this protein.

The phosphorylation of clock proteins plays a critical role in generating proper circadian rhythms (Lu et al., 2011). Overexpression of CK2 regulatory subunits (CKB3 or CKB4) in *Arabidopsis* displays increased CK2 activity, a reduction of the subjective day length inducing alterations in clock-regulated gene expression, hypocotyl elongation, and flowering time (Sugano et al., 1999; Perales et al., 2006). CCA1 and LHY are phosphorylated by CK2 and this phosphorylation is required for the normal functioning of the CCA1 protein (Daniel et al., 2004). CK2 is involved in the temporal regulation of CCA1 protein activity, targeting it for degradation, promoting CCA1 dimerization, CCA1-DNA complex formation, and interaction with the promoters of downstream genes, such as TOC1 (Kusakina and Dodd, 2012).

Thus, increasing levels of ABA lead to an increase in TOC1 levels, resulting in the repression of the ABA signal through the down-regulation of ABAR/CHLH/GUN5 and CCA1 by TOC1. Concomitantly, CK2 activity would regulate the level of CCA1 repression through its controlled degradation, and regulation of protein and DNA interaction, in a process analogous to the SnRK2 repression explained earlier (Figure 1B).

## Concluding Remarks

Our understanding of ABA signaling has expanded exponentially in recent years. Two seminal works on a family of soluble proteins that are able to bind ABA made possible the construction of a functional model for ABA signal transduction (Ma et al., 2009; Park et al., 2009). These ABA receptors (PYR/PYL/RCAR), together with SnRK2 kinases and PP2C phosphatases constitute the central core of ABA signaling.

The central core of ABA signaling controls a fast cellular response to ABA that ranges from activation of ion transports to a large transcription reprogramming. Nevertheless, there is growing evidence that, following the initial response to ABA, the persistence of the signal results in a secondary response that leads to stress adaptation. ABA signaling is also capable of incorporating several other processes, such as circadian rhythms, in their output.

Protein phosphorylation and dephosphorylation play a central role in ABA signaling and promote the activation, deactivation, sequestration and degradation of a wide range of protein regulators. In addition to protein phosphorylation, regulation of protein stability by the *26S* proteasome is an important mechanism for ABA signaling.

ABA signaling appears to undergo dynamic changes in the steady state of some of its major components (Figure 1). In the absence of the hormone, the PYR/PYL/RCAR receptors, the SnRK2 kinases, and several transcription factors that elicit ABA response are degraded by the proteasome, and/or inactivated. This results in an effective dampening of the ABA signal. Conversely, ABA has a protecting effect on the protein turnover of these components and their activation. At the same time, ABA transcriptionally regulates the future changes in the ABA signal.

CK2 mediated stabilization and destabilization of proteins represents a known evolutionarily conserved mechanism. Phosphorylation by CK2 enhances the polyubiquitination of target proteins, signaling to or protecting from proteasomal degradation. For instance, CK2 phosphorylation regulates photomorphogenesis stabilizing HY5 and HR1 and promoting degradation of PIF1 (Hardtke et al., 2000; Park et al., 2008; Bu et al., 2011). In addition, CK2 does not appear to be under major transcriptional regulation and the holoenzyme activity appears to always be in an “on” state. These characteristics make CK2 a housekeeping kinase that can modify protein functions and protein turnover in a dynamic way. In the context of ABA signaling, CK2 is already known to promote SnRK2 degradation through the *26S* proteasome and inactivation through the interaction with PP2C, and has been connected with ABAR phosphorylation. Exploring the effects of CK2 in the phosphorylation and ubiquitination of other ABA regulators should help to give a broader perspective on ABA signal, protein stability and integration of other processes in abiotic stress responses.

### Conflict of Interest Statement

The authors declare that the research was conducted in the absence of any commercial or financial relationships that could be construed as a potential conflict of interest.
